# Magnetic Temperature-Sensitive Solid-Lipid Particles for Targeting and Killing Tumor Cells

**DOI:** 10.3389/fchem.2020.00205

**Published:** 2020-04-09

**Authors:** Małgorzata Świętek, Rostyslav Panchuk, Nadia Skorokhyd, Peter Černoch, Nataliya Finiuk, Olha Klyuchivska, Martin Hrubý, Matúš Molčan, Walter Berger, Jirí Trousil, Rostyslav Stoika, Daniel Horák

**Affiliations:** ^1^Institute of Macromolecular Chemistry of the Czech Academy of Sciences, Prague, Czechia; ^2^Department of Regulation of Cell Proliferation and Apoptosis, Institute of Cell Biology, National Academy of Science of Ukraine, Lviv, Ukraine; ^3^Institute of Experimental Physics, Slovak Academy of Sciences, Košice, Slovakia; ^4^Department of Medicine I, Medical University of Vienna, Institute of Cancer Research and Comprehensive Cancer Center, Vienna, Austria

**Keywords:** magnetic, temperature sensitive, solid lipid particles, human leukemia cells, doxorubicin- and vincristine-resistant sublines, fluorescent microscopy, FACS analysis, ROS production

## Abstract

Magnetic and temperature-sensitive solid lipid particles (mag. SLPs) were prepared in the presence of oleic acid-coated iron oxide (IO-OA) nanoparticles with 1-tetradecanol and poly(ethylene oxide)-*block*-poly(ε-caprolactone) as lipid and stabilizing surfactant-like agents, respectively. The particles, typically ~850 nm in hydrodynamic size, showed heat dissipation under the applied alternating magnetic field. Cytotoxic activity of the mag.SLPs, non-magnetic SLPs, and iron oxide nanoparticles was compared concerning the mammalian cancer cell lines and their drug-resistant counterparts using trypan blue exclusion test and MTT assay. The mag.SLPs exhibited dose-dependent cytotoxicity against human leukemia cell lines growing in suspension (Jurkat and HL-60/wt), as well as the doxorubicin (Dox)- and vincristine-resistant HL-60 sublines. The mag.SLPs showed higher cytotoxicity toward drug-resistant sublines as compared to Dox. The human glioblastoma cell line U251 growing in a monolayer culture was also sensitive to mag.SLPs cytotoxicity. Staining of U251 cells with the fluorescent dyes Hoechst 33342 and propidium iodide (PI) revealed that mag.SLPs treatment resulted in an increased number of cells with condensed chromatin and/or fragmented nuclei as well as with blebbing of the plasma membranes. While the Hoechst 33342 staining of cell suggested the pro-apoptotic activity of the particles, the PI staining indicated the pro-necrotic changes in the target cells. These conclusions were confirmed by Western blot analysis of apoptosis-related proteins, study of DNA fragmentation (DNA laddering due to the inter-nucleosomal cleavage and DNA comets due to single strand breaks), as well as by FACS analysis of the patterns of cell cycle distribution (pre-G1 phase) and Annexin V/PI staining of the treated Jurkat cells. The induction of apoptosis or necrosis by the particles used to treat Jurkat cells depended on the dose of the particles. Production of the reactive oxygen species (ROS) was proposed as a potential mechanism of mag.SLPs-induced cytotoxicity. Accordingly, hydrogen peroxide and superoxide radical levels in mag.SLPs-treated Jurkat leukemic cells were increased by ~20–40 and ~70%, respectively. In contrast, the non-magnetic SLPs and neat iron oxides did not influence ROS levels significantly. Thus, the developed mag.SLPs can be used for effective killing of human tumor cells, including drug-resistant ones.

## Introduction

Cancer is one of the top causes of death globally, with 17 million new cases and 9.6 million deaths worldwide in the last year (Cancer Research UK, 2019). Apart from surgery and radiotherapy, chemotherapy remains currently a leading cancer treatment option. High cytotoxicity of anticancer drugs especially toward rapidly dividing cells is the key factor of the therapeutic efficacy; however, simultaneously, due to the lack of specificity, the origin of numerous adverse effects is one of the major chemotherapy disadvantages (Palumbo et al., 2013). Many short and long-term drawbacks associated with chemotherapy significantly hinder the therapeutic process and additionally can diminish the life quality of patients in remission. Beside the lack of specificity of anticancer agents, the rapid development of drug resistance in tumor cells is the second major limitation of chemotherapy that greatly reduces its effectiveness (Housman et al., 2014). For this reason, massive effort has been made to increase the selectivity and reduce the probability of resistance development to improve safety and efficacy of chemotherapeutic agents.

Progress in physics, chemistry, materials science, and biology achieved over the last decades, led to elaboration of new strategies in drug delivery systems based on micro- and nanoscale carriers. Surface modification with biological moieties, such as antibodies and peptides, enabled their enhanced delivery to the targeting site and limited their rapid clearance by the immune system (Myung et al., 2017; Smith and Gambhir, 2017; Zhu et al., 2017; Bayda et al., 2018; Kim et al., 2018; Palazzolo et al., 2018; Singh et al., 2019; Zottel et al., 2019). Various polymers were used to produce microcapsules, liposomes, and micelles that are able to enclose both hydrophilic and hydrophobic drugs, protect them from severe conditions, and release them in controllable manner at the final site of destination (Singh et al., 2010; Akbarzadeh et al., 2013; Zhang et al., 2014). In addition to polymers, also surfactant-stabilized lipids, represented, e.g., by fatty acids, glycerides, and waxes, were used to develop vehicle-like drug carriers called solid-lipid particles (SLPs) that remain in solid state at room temperature (RT) (Muñoz de Escalona et al., 2016; Mishra et al., 2018). The SLPs are characterized by biodegradability, biocompatibility, and higher entrapment efficiency compared to liposomes (Albuquerque et al., 2015; Brezaniova et al., 2016). A further advantage of SLPs is the easiness of their scale-up synthesis and sterilization, and a more affordable prize than, e.g., for polymer nanoparticles.

Moreover, different types of nanoparticles, including iron oxide, gold, gadolinium, and doped NaYF_4_ upconversion particles, were conjugated with biologically active molecules to produce multi-functional platforms, that combine therapeutic effect with a possibility of, e.g., tissue imagining (Liong et al., 2008; Li et al., 2016; Kostiv et al., 2017). Among these materials, only iron oxide and gadolinium nanoparticles possess magnetic properties, which enable controlling their movement remotely through an external magnetic field (Truzzi et al., 2017). This feature makes them very useful for various elaborated biomedical applications including cell labeling and separation, non-invasive magnetic resonance imagining (MRI), magnetically modulated drug delivery systems, regenerative medicine, and cancer therapy (Mody et al., 2014; Estelrich et al., 2015; Plouffe et al., 2015; Świętek et al., 2019a). In cancer treatment, iron oxide nanoparticles can serve as a drug carrier or source of heat under alternating magnetic field in the local fluid hyperthermia approach (Périgo et al., 2015). In contrast to non-cancerous tissue, tumors are characterized by deficient vascularity, which is not able to ensure effective heat exchange. Consequently, an increased temperature in the range of 41–46°C leads to rapid cancer cell overheating and apoptosis induction. While iron oxide nanoparticles are generally perceived as highly biocompatible and safe materials, cytotoxicity may be caused by generation of radical oxygen species (ROS) mediated by Fenton reaction (Wydra et al., 2015). A disruption of ROS balance elicits an oxidative stress in cells and leads to harmful damages of carbohydrates, DNA, lipids, and proteins (Abakumov et al., 2018). Therefore, an excessive amount of ROS induced by the iron oxide nanoparticles can be used as an effective non-chemotherapeutic strategy to treat tumors (Shen et al., 2018; Sun et al., 2018).

Given this background, the aim of this report was to develop and characterize a new type of the magnetic temperature-sensitive SLPs (mag.SLPs) intended for cancer treatment. We demonstrated that the mag.SLPs were capable of effective killing of human tumor cells, including their drug-resistant sublines, even without a conjugated anticancer drug. The anticancer effect of the mag.SLPs was comparable to that of doxorubicin (Dox), a cornerstone and gold standard in the anticancer chemotherapy (Lippens et al., 1998; Minotti et al., 2004; Zhao et al., 2017).

## Experimental

### Materials

FeCl_2_ · 4H_2_O, FeCl_3_ · 6H_2_O, 1-tetradecanol (TD), Dulbecco's modified Eagle's culture medium (DMEM), Hoechst 33342, and propidium iodide (PI) were purchased from Sigma-Aldrich (St. Louis, MO, USA); 3-(4,5-dimethylthiazol-2-yl)-2,5-diphenyltetrazolium bromide (MTT) was from Biomedica (Vienna, Austria). Twenty five percent NH_4_OH, 70% oleic acid (OA), dichloromethane (DCM), and methylene chloride were obtained from Lach-Ner (Neratovice, Czech Republic). Trypan blue DV-T10282 was from Life Technologies (Carlsbad, CA, USA). Doxorubicin (Dox) was purchased from Actavis (Bucharest, Romania), vincristine was from Teva (Haarlem, Netherlands), Roswell Park Memorial Institute (RPMI) culture medium and fetal bovine serum (FBS) were from APP (Vienna, Austria). All other chemicals were purchased from Sigma-Aldrich. Poly(ethylene oxide)-*block*-poly(ε-caprolactone) (BL; *M*_n_ = 6,500 Da, *M*_w_ = 7,100 Da, polydispersity = 1.1) was synthesized by a ring-opening polymerization of ε-caprolactone initiated by poly(ethylene oxide) monomethyl ether (degree of polymerization = 45), as previously described (Trousil et al., 2019). Ultrapure water for the magnetic particle synthesis was obtained from a Milli-Q Gradient A10 system (Millipore; Molsheim, France).

### Preparation of Iron Oxide Nanoparticles Stabilized With Oleic Acid (IO-OA)

Iron oxide (IO) nanoparticles were prepared by coprecipitation method according to a previously published protocol, with slight modifications (Świętek et al., 2019b). Briefly, FeCl_2_ · 4H_2_O (1.191 g) and FeCl_3_ · 6H_2_O (3.242 g) were dissolved in water (175 ml) and the solution was heated at 70°C for 10 min. Twenty five percent ammonia solution (10 ml) was diluted to 35 ml and added dropwise to the above solution under precipitation of black IO nanoparticles. The mixture was heated at 90°C for 1 h, cooled to RT, and cautiously transferred in 3% OA/DCM solution (v/v). Resulting IO-OA particles were washed with DCM four times (50 ml each), redispersed in DCM with sonication (UW 3100 sonicator; Bandelin Electronic; Berlin, Germany) for 5 min at 30% amplitude to reach concentration of 4 mg/ml, and used for preparation of mag.SLPs. For biological experiments, aqueous IO-OA particle dispersion was prepared by gradual transfer of the particles from DCM to water via ethanol/water mixtures using magnetic separation.

### Preparation of Magnetic Solid-Lipid Particles (mag.SLPs)

Mag.SLPs were prepared according to an earlier procedure (Brezaniova et al., 2016). Briefly, TD (200 mg) and IO-OA (20 mg) dispersion in DCM (5 ml) were mixed and DCM was evaporated at 50°C. Solution of BL (50 mg) in DCM (2 ml) and water (5 ml) were heated at 40°C and added into the system, the mixture was homogenized with a T25 digital Ultra-Turrax high-speed homogenizer (IKA; Staufen, Germany) at 12,000 rpm for 10 min, and subsequently rehomogenized with a GM 2070 probe-type sonicator (Bandelin Electronic) by continuous sonication for 6 min (80% amplitude). After the homogenization, the formulation was stored on ice bath for at least 30 min, transferred into a clean vial and the volume was adjusted with water to 5 ml. The dispersion was stored in the fridge at 4°C. Non-magnetic SLPs were prepared analogously, but in the absence of IO-OA.

### Particle Characterization

Transmission electron microscopy was used to measure the number-average particle diameter (*D*_n_) and the particle size distribution (dispersity Ð) using a Fei Tecnai G2 Spirit TEM microscope (Brno, Czech Republic). The size of mag.SLPs was evaluated using a MAIA3 scanning electron microscope (Tescan; Brno, Czech Republic). Dynamic light scattering (DLS) of aqueous SLP dispersions (25°C) on a Zen 3600 Zetasizer (Malvern Instruments; Malvern, UK) was used to determine ζ-potential, hydrodynamic diameter (*D*_h_), and polydispersity index (*PDI*) from the intensity distribution curve. Iron content was determined using a Perkin Elmer 3110 atomic absorption spectrometer (AAS; Waltham, MA, USA), content of C was measured on a FlashSmart^TM^ elemental analyzer (Thermo Fisher Scientific; Waltham, MA, USA), and thermogravimetric analysis was performed in air flow with 10°C/min heating rate on a Perkin Elmer TGA 7 thermogravimetric analyzer (Waltham, MA, USA) equipped with Pyris software. The heat effect of the mag.SLPs was tested under an alternating magnetic field (AMF) by homemade AC system and temperature changes were monitored by a fiber optic temperature sensor (FISO Technologies; Québec, Canada) (Skumiel et al., 2016). Magnetization of dry particles was determined using an EV9 vibrating sample magnetometer (DSM Magnetics, ADE Corp.; Lowell, MA, USA) at RT.

### Biological Experiments

#### Cell Culture

Human myeloid leukemia HL-60/wt cells, their multidrug resistant sublines selected against Dox (HL-60/adr with multidrug resistance-associated protein 1 (MRP1) = ABCC1 over-expression) or vincristine (HL-60/vinc with P-glycoprotein = ABCB1 over-expression), and human leukemia Jurkat T-cells were obtained from a collection of the Institute of Cancer Research and Comprehensive Cancer Center (Medical University of Vienna, Austria). Human glioblastoma U251 cells were obtained from a collection of the Institute of Molecular Biology and Genetics NASU (Kiev, Ukraine). Cells were cultivated in complete RPMI-1640 or DMEM culture medium supplemented with 10% FBS at 37°C under 5% CO_2_ atmosphere. Drug-resistant HL-60/adr cells were cultured in RPMI-1640 medium supplemented with 10% FBS and 0.1 μM doxorubicin at 37°C in 5% CO_2_ atmosphere. Prior the experiments, HL-60/adr cells were cultured in drug-free medium for 2 weeks (Ke et al., 2008). HL-60/vinc cells were cultured in RPMI-1640 medium supplemented with 10% FBS and 0.1 μM vincristine at standard conditions. Reverse transcription polymerase chain reaction (RT-PCR) was performed to determine MRP1 mRNA. P-glycoprotein level was examined by the Western blot analysis at the Institute of Cancer Research of Medical University of Vienna.

#### MTT Assay for Determination of Cell Viability

MTT assay based on the colorimetric measurement of formazan formed after reduction of MTT by cellular NAD(P)H-dependent oxidoreductases was used for examination of the antineoplastic activity of the particles. Briefly, the cells were seeded into 96-well plates in 100 μl of complete culture medium at a concentration of 3,000 substrate-dependent cells or 15,000 cells per well, and left incubated overnight as described above. The formulations to be tested (100 μl aliquots) were added to the culture medium at different concentrations and left incubated for 24 or 72 h. The MTT assay was performed in accordance with the manufacturer's recommendations and assessed using an EL ×800 absorbance reader (BioTek Instruments; Winooski, VT, USA). The results of the MTT assay were expressed as a percentage relative to the control cells in the medium considered to be 100%. The half maximal inhibitory concentration (*IC*_50_) of the tested particles was calculated as the concentration that reduced cell vitality by 50%.

#### Trypan Blue Exclusion Test for Determination of Cytotoxicity

Cells were seeded into 24-well plates in 1 ml of complete culture medium at concentrations of 100,000 of adherent and 1 million of suspension cells per well and incubated overnight as described above. The cells were treated with different amounts of the particles or Dox for 24 or 72 h and a trypan blue exclusion test, based on the penetration of the dye into the dead cells was used for the cytotoxicity measurement. Number of alive (unstained) cells was counted in a Neubauer hemocytometer chamber (Thermo Fisher Scientific) 2 min after addition of trypan blue (0.05% of final concentration).

#### Determination of ROS in Cells

Jurkat cells (1 ml/well) were seeded in 24-well plates at concentration of 1 million of cells per well, cultured in the presence of the particles for 24 h, and ROS level was measured. Briefly, the cells were incubated with 10 μM dihydroethidium (DHE) to detect superoxide radicals in FL2 channel before analysis or with 1 μM 2′,7′-dichlorofluorescein diacetate (DCFDA) added 30 min before treatment of the cells to detect hydrogen peroxide in FL1 channel. The cells were analyzed using a FACScan flow cytometer (BD Biosciences; San Jose, CA, USA) and Summit v3.1 software (Cytomation; Fort Collins, CO, USA). The results of the FACS analysis of cells stained with DCFDA or DHE were expressed as a percentage relative to the control cells in the medium (100%).

#### Microscopic Analysis of Cell Morphology

Human glioblastoma U251 cells were seeded at concentration of 100,000 cells/well on glass microscopic slides located in 12-well plates and cultured for 24 h in the presence of the particles. Subsequently, the cells were double stained with Hoechst 33342 (0.2–0.5 μg/ml) and PI (20 μg/ml) to discriminate alive, apoptotic, and necrotic cells. Chromatin material of nucleus of the treated cells was stained with the DNA-specific blue-fluorescent dye Hoechst 33342 (λ_Ex_/λ_Em_ (with DNA) = 352/461 nm). The dead cells uptake the DNA/RNA-specific red-fluorescent dye PI (λ_Ex_/λ_Em_ (with DNA) = 535/617 nm) due to a damage of their plasma membrane (Atale et al., 2014). The cells were incubated additionally for 20–30 min in the presence of Hoechst 33342 dye. PI was added immediately before the cell examination under a Carl Zeiss fluorescent microscope (Oberkochen, Germany) equipped with an AxioImager A1 camera (400 × magnification) and Image-Pro7.0 software (Media Cybernetics; Rockville, MD, USA) (Finiuk et al., 2017).

#### Western Blot Analysis of Apoptosis-Related Proteins

Jurkat cells were seeded in 24-well plates at concentration of 10^6^ cells per well (1 ml/well), cultured for 24 h in the presence of the particles (1 and 5 μg/ml), and the level of apoptosis-related protein (cleaved poly(ADP-ribose) polymerase 1—PARP1) was measured. Cellular proteins were extracted with a lysis buffer (20 mM Tris-HCl, pH 8.0, 1% Triton-X100, 150 mM NaCl, 50 mM NaF, 0.1% sodium dodecyl sulfate—SDS) that contained 1 mM phenylmethanesulfonyl fluoride and 10 μg/ml of cOmplete™ protease inhibitor cocktail (Roche; Basel, Switzerland). Proteins (30 μg/lane) were separated by SDS/PAGE gel-electrophoresis and transferred onto a poly(vinylidene difluoride) membrane (Senkiv et al., 2016). Primary antibodies for cleaved PARP1 (Asp214), catalog number D64E10, were purchased from Cell Signaling Technology (Danvers, MA, USA) and those for beta-actin AC-15 monoclonal from Sigma-Aldrich. Secondary peroxidase-conjugated antibodies (Cell Signaling Technology) were applied at final dilution of 1:5,000. Enhanced chemiluminescence detection reagents (Sigma-Aldrich) were used for protein visualization. Densitometric analysis of the protein level was performed using open source ImageJ software.

#### FACS Analysis of Cell Death

##### Apoptosis analysis

For this analysis, cells were stained with FITC-conjugated Annexin V and propidium iodide (PI) using an apoptosis detection kit (BD Biosciences; San Jose, CA, USA) according to the manufacturer's instructions. Briefly, 24 h after the addition of particles (1, 2.5, and 5 μg/ml), Jurkat T-leukemia cells were centrifuged at 2,000 rpm, washed twice with 1 × PBS, and incubated for 15 min in Annexin V binding buffer (BD Pharmingen; USA) containing 1/50 volume of FITC-conjugated Annexin V solution and PI (50 μg/ml). The samples were diluted two times by appropriate volume of Annexin V binding buffer and immediately measured on FL1/FL2 (FITC-PI) channels of a FACScan flow cytometer (Becton Dickinson; Franklin Lakes, NJ, USA). Analysis was carried out using Cytomation Summit Software v3.1.

##### Cell cycle analysis

Cell cycle analysis was assessed according to Walker et al. (1993). After treatment with particles (1, 2.5, and 5 μg/ml), the cells (2 × 10^6^) were collected, pelleted by spinning (1,000 rpm) at 4°C for 5 min, resuspended in cold 1 × PBS (1 ml), and fixed by dropwise addition of absolute ethanol (4 ml) at −20°C. On the next day, fixed cells were centrifuged and the cell pellet was resuspended in PBS (1 ml). DNase-free RNase A (100 μl; 200 μg/ml; Invitrogen, USA) was added to the cell suspension that was incubated at 37°C for 30 min. PI (100 μl; 1 mg/ml) was added to the samples incubated at room temperature for 5–10 min, which were then analyzed on a FACScan flow cytometer. Cell cycle analysis was carried out using Cytomation Summit Software v3.1.

#### Analysis of DNA Fragmentation by Electrophoresis in Agarose Gel

Jurkat cells (10^6^ cells per well; 1 ml/well) were seeded in 24-well plates and cultured for 24 h in the presence of the particles (1 and 5 μg/ml) or doxorubicin (0.25 μg/ml). DNA was extracted, as described by (Liu et al., 2016). The cells were harvested, washed with PBS, and the pellets were lysed with 50 μl of lysis buffer (1% NP-40, 20 mM EDTA, 50 mM Tris-HCl; pH 7.5) for 10 min. After centrifugation at 7,000 rpm for 10 min, the supernatants were prepared in an equal volume of 1% sodium dodecyl sulfate, incubated with RNase A (2.5 mg/ml) at 37°C for 2 h, which was followed by digestion with proteinase K (2.5 mg/ml) at 37°C for 2 h. After the addition of 0.5 volumes of 10 M ammonium acetate, DNA was precipitated with 2 volumes of cold isopropyl alcohol and collected by centrifugation (13,000 rpm; 25 min). DNA was dissolved in TAE buffer (Tris-acetate-EDTA), separated by electrophoresis in 1.5% agarose containing 0.005% ethidium bromide, and visualized under UV light.

#### DNA Comet Assay (Single Cell Gel Electrophoresis)

DNA comet assay was performed under alkaline conditions, as described by Liao et al. (2009). Jurkat cells (200,000 cells in 1 ml) were seeded in 24-well plates and treated with the particles (1, 2.5, and 5 μg/ml) or doxorubicin (0.25 μg/ml) for 24 h. After that, slides were stained with ethidium bromide (10 μg/ml; Sigma-Aldrich) and observed using a Zeiss fluorescent microscope with an AxioImager A1 camera. CASPLab-1.2.2 software (Wroclaw, Poland) was used for calculation of the percentage of tail DNA in the nuclei of 200 cells per each sample.

#### Statistical Analysis of Experimental Data

The results were analyzed using ANOVA test with Prism 6 software (GraphPad; La Jolla, CA, USA) and presented as the mean ± standard deviation of three replications in two parallels (*n* = 6). The results of FACS experiments were analyzed by one-way analysis of variance ANOVA, followed by Tukey's *post hoc* multiple comparison test. Statistical significance was identified at *p* ≤0.05.

## Results

### Iron Oxide Nanoparticles

**Figure 1:**
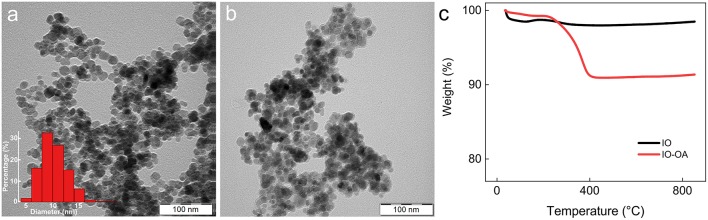
TEM micrographs of **(a)** IO (particle size distribution inserted) and **(b)** IO-OA nanoparticles. **(c)** Thermogravimetric analysis of IO and IO-OA nanoparticles.

Starting hydrophilic iron oxide (IO) nanoparticles were prepared by a simple coprecipitation technique in water (Świętek et al., 2019b). As the SLPs are lipophilic, the IO particles had to be rendered hydrophobic to make their encapsulation possible. The hydrophilic IO was therefore hydrophobized with OA serving as an efficient steric stabilizer and enabling IO-OA particles to be transferred in DCM and dispersed in the lipid phase. Synthesized IO had a spheroidal shape and was moderately polydisperse in size (Ð = 1.2) with the number-average diameter *D*_n_ = 10 nm according to TEM (Figure 1a). Hydrodynamic diameter of the IO particles was *D*_h_ = 150 nm with *PDI* = 0.17 and ζ-potential = 14 mV (Table 1). TGA of the IO nanoparticles revealed the total weight loss <1.5 wt.% mainly up to 100°C (Figure 1c). Magnetic measurements then showed that the particles had saturation (*M*_s_) and remanent magnetization (*M*_r_), and coercivity (*H*_c_) of 53.1 and 0.9 A · m^2^/kg, and 6.5 Oe, respectively (Table 1).

**Table 1:** Composition and properties of the particles.

**Particles**	**C**	**Fe**	***D*_**h**_**	***PDI***	**ζ-potential**	***M*_**s**_**	***M*_**r**_**	***H*_**c**_**
	**(wt.%)**	**(wt.%)**	**(nm)**		**(mV)**	**(A · m^**2**^/kg)**	**(A · m^**2**^/kg)**	**(Oe)**
IO	–	73.1	150	0.17	14	53.1	0.9	6.5
IO-OA	2.5	67.1	244	0.33	15	49.8^a^	0.9^a^	7.9^a^
SLPs	73.5	–	866	0.48	−30	–	–	–
Mag.SLPs	69.7	5.6	854	0.59	−37	3.6	0.1	4.3

a *In DCM. Final volume of the aqueous SLP dispersion was 5 ml, mag.SLPs contained 20 mg of IO*.

*IO, iron oxide; IO-OA, oleic acid-stabilized iron oxide; SLPs, solid lipid particles; mag.SLPs, magnetic solid-lipid particles; D_h_, hydrodynamic diameter (DLS); PDI, polydispersity index (DLS); M_s_, saturation magnetization; M_r_, remanent magnetization; and H_c_, coercivity*.

In contrast to the IO, TEM of the IO-OA showed the presence of an additional halo layer around the nanoparticles (Figure 1b). Adsorption of OA on the IO surface was also confirmed by DLS and TGA (Figure 1c; Table 1). *D*_h_ of IO-OA (244 nm) was nearly by 100 nm larger than that of IO, which was also associated with increase in *PDI* to 0.33, albeit the ζ-potential of IO-OA (15 mV) was almost the same as that of IO. According to TGA and elemental analysis of IO-OA particles, the total weight loss was 8.6 wt.% up to 800°C and content of C reached 2.5 wt.% (Figure 1c; Table 1). The IO-OA nanoparticles exhibited a similar magnetic behavior as the IO, i.e., *M*_s_, *M*_r_, and *H*_c_ was 49.8 and 0.9 A · m^2^/kg and 7.9 Oe, respectively (Table 1).

### Magnetic Solid-Lipid Particles (SLPs)

**Figure 2:**
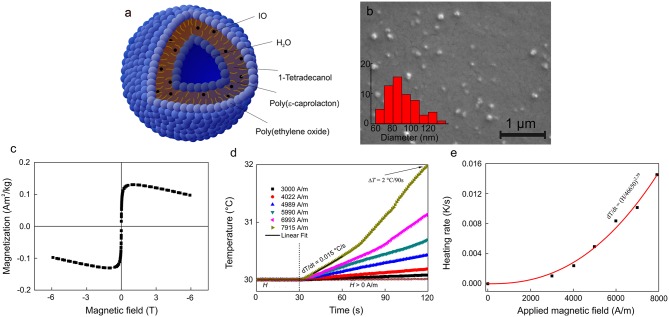
**(a)** Schematic view and **(b)** SEM micrograph of mag.SLPs (particle size distribution inserted). **(c)** Magnetization curve, **(d)** dependence of temperature on time, and **(e)** heating rate of mag.SLP dispersion (12.5 mg of iron oxide per ml) as a function of the applied magnetic field. **(c)** Measured at 298 K, **(d)** after exposition to AMF = 3–7.9 kA/m and *f* = 187 kHz, and **(e)** *f* = 187 kHz.

SLPs were obtained by a hot high-performance homogenization followed by ultrasonication above the melting point of the lipid phase (Figure 2a). According to DLS, *D*_h_, *PDI*, and ζ-potential of the SLPs were 866 nm, 0.48, and 30 mV, respectively (Table 1). The incorporation of IO-OA in the SLPs exerted almost no influence on *D*_h_ (854 nm), but slightly decreased ζ-potential (by −7 mV); both parameters did not significantly change after 3 weeks of storage at 5°C, documenting the good stability of the particles. SEM micrograph of the mag.SLPs revealed spherical morphology with the diameter *D*_n_ = 60–140 nm (Figure 2b); majority of the particles (~30%) had *D*_n_ = 70–90 nm, and the particles <70 nm and >100 nm accounted for 5 and 16% of the total, respectively. The mag.SLPs exhibited weak ferrimagnetic magnetic behavior originating from IO-OA (Figure 2c; Table 1). The *M*_s_, *M*_r_, and *H*_c_ were 3.6 and 0.1 A · m^2^/kg, and 4.3 Oe, respectively. Iron and carbon content in the mag.SLP was of 5.6 and 69.7 wt.%, respectively, while the carbon amount in the non-magnetic SLPs was 73.5 wt.%. To prove the heating principle, helping to dissolve the lipid capsule around the iron oxide, temperature increase of the mag.SLPs containing a higher content of Fe (28.8 wt.%) was investigated under AMF with various field intensity *H* up to 7.9 kA/m and frequency *f* = 187 kHz applied for 90 s (Figure 2d). The mag.SLPs were heated up by 2°C in 90 s at the highest *H* = 7.9 kA/m. In contrast, the lowest *H* = 3 kA/m caused only negligible thermic effect.

### Cytotoxicity Evaluation of the Particles

**Figure 3:**
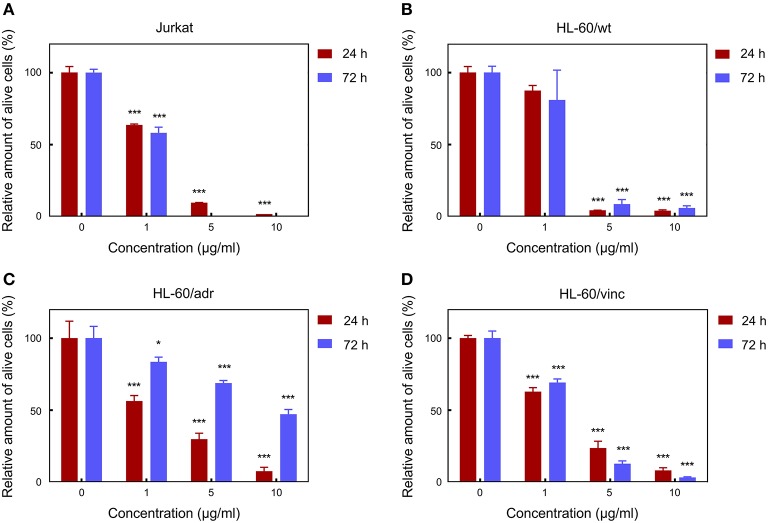
Cytotoxicity of the mag.SLPs against **(A)** T leukemia Jurkat cells, **(B)** human myeloid leukemia HL-60/wt cells, **(C)** human myeloid leukemia HL-60/adr cells resistant to doxorubicin, and **(D)** human myeloid leukemia HL-60/vinc cells resistant to vincristine determined by trypan blue exclusion test on 24 and 72 h. *p ≤ 0.05 and ***p ≤ 0.001 compared to the non-treated control cells.

The potential of mag.SLPs as an alternative to cytotoxic anticancer drugs was initially evaluated *in vitro* toward four cell types growing in suspension, i.e., T-cell leukemia Jurkat cells, human myeloid leukemia HL-60/wt cells, and drug-resistant leukemia HL-60/adr and HL-60/vinc sublines exhibiting a multidrug resistance phenotype induced by selection against Dox and vincristine, respectively, using the trypan blue exclusion test (Figure 3). Mag.SLPs exhibited a distinct dose dependent cytotoxicity. Significant time dependence (24 *vs*. 72 h) was observed only for HL-60/adr cells (Figure 3), where cytotoxicity was lower after 72 h than that after 24 h, probably due to a renewal of cell proliferation at a longer treatment time. Nevertheless, the exact reason of higher resistance of the Dox-resistant cells of HL-60/adr subline to cytotoxic activity of the particles after 72 h of treatment compared to that after 24 h needs additional investigation. *IC*_50_ for the mag.SLPs applied for 24 h was 3.8, 2.7, 1.9, and 2.3 μg/ml for Jurkat, HL-60/wt, HL-60/adr, and HL-60/vinc cells, respectively (Table 2). Incubation of Jurkat, HL-60/wt, drug-resistant HL-60/adr and HL-60/vinc cells with the mag.SLPs for 72 h resulted in *IC*_50_ = 0.9, 2.3, 9.3, and 2.4 μg/ml, respectively. For Jurkat and HL-60/wt cells, *IC*_50_ was significantly higher compared to *IC*_50_ for Dox that was 0.3 μg/ml for Jurkat T-leukemia and 0.6 μg/ml for and HL-60 leukemia cells (24 h of treatment). In contrast, drug-resistant sublines were more sensitive to the mag.SLPs than to Dox. *IC*_50_ for the mag.SLPs for HL-60/adr subline was 3.2 times lower (1.9 μg/ml) than for Dox (6 μg/ml). For HL-60/vinc, *IC*_50_ for the mag.SLPs was also 1.3 times lower (2.3 μg/ml) compared to *IC*_50_ for Dox (3 μg/ml).

**Table 2:** Half maximal inhibitory concentration (*IC*_50_) for various particles and doxorubicin (Dox) toward several tumor cell lines determined by trypan blue exclusion test.

	**Cells**	***IC***_****50****_ **(μg/ml)**				
		**Mag.SLPs**	**SLPs**	**IO-OA**	**IO**	**Dox**
Jurkat	24 h	3.8	>10	>10	>10	0.3
	72 h	0.9	10	>10	>10	0.1
HL-60/wt	24 h	2.7	3.5	3.5	0.9	0.6
	72 h	2.3	3.3	1.2	0.9	0.1
HL-60/adr	24 h	1.9	–	–	–	6.0
	72 h	9.3	–	–	–	3.6
HL-60/vinc	24 h	2.3	–	–	–	3.0
	72 h	2.4	–	–	–	0.8
U251	24 h	1.2	1.1	7.4	5.9	1.7
	72 h	2.3	1.6	1.3	3.4	0.6

–*Not determined*.

**Figure 4:**
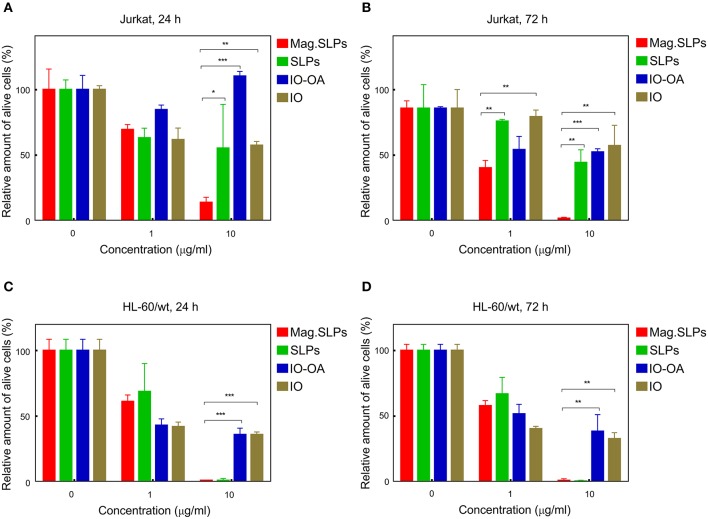
Cytotoxicity of mag.SLPs, SLPs, IO-OA, and IO particles against **(A,B)** T leukemia Jurkat cells and **(C,D)** human myeloid leukemia HL-60/wt cells determined by trypan blue exclusion test after **(A,C)** 24 and **(B,D)** 72 h of incubation. *p ≤ 0.05, **p ≤ 0.01, and ***p ≤ 0.001 compared to the mag.SLPs.

**Figure 5:**
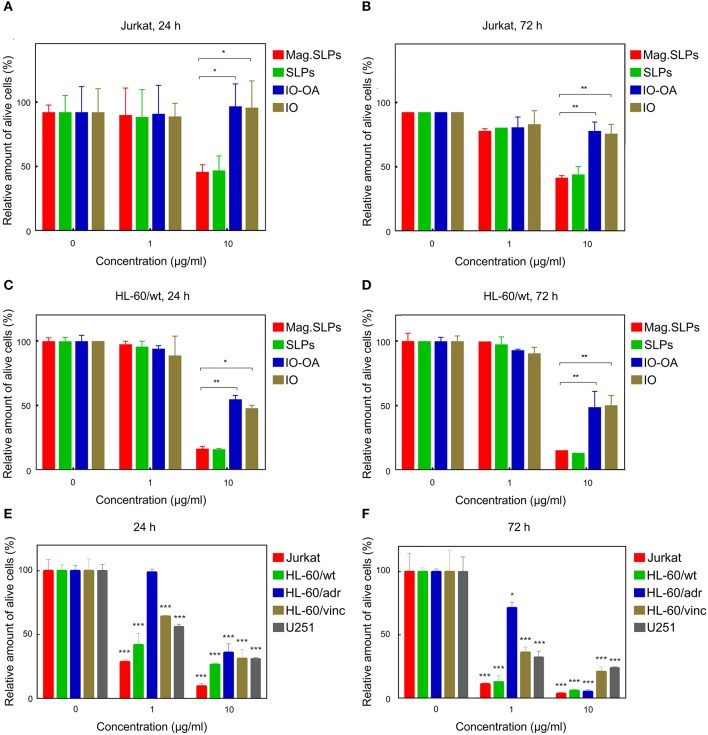
Cytotoxicity of mag.SLPs, SLPs, IO-OA, and IO particles against **(A,B)** T leukemia Jurkat cells and **(C,D)** human myeloid leukemia HL-60/wt cells determined by MTT assay after **(A,C)** 24 and **(B,D)** 72 h of incubation. **(E,F)** Cytotoxicity of Dox toward T leukemia Jurkat cells, human myeloid leukemia HL-60/wt cells, human myeloid leukemia HL-60/adr cells resistant to Dox, human myeloid leukemia HL-60/vinc cells resistant to vincristine, and human glioblastoma U251 cells determined by trypan blue exclusion test after **(E)** 24 and **(F)** 72 h of incubation. *p ≤ 0.05, **p ≤ 0.01, and ***p ≤ 0.001 compared to non-treated control cells.

Mag.SLPs are a complex system made of several components, including lipid coat or IO, that can participate in the cytotoxic activity. To address origin of this activity, cytotoxic effects of the mag.SLPs, SLPs, IO-OA, and IO toward Jurkat T-cells and HL-60/wt cells after 24 and 72 h of treatment were compared using both trypan blue exclusion test and MTT assay (Figures 4, 5). For Jurkat cells, the IO, IO-OA and SLPs were much less cytotoxic in trypan blue exclusion test than the mag.SLPs, especially at the highest concentration of 10 μg/ml (Figures 4A,B). Moreover, when T-leukemia cells of Jurkat line were targeted with the IO-OA, no cytotoxic effect was detected after 24 h of incubation and only moderate cytotoxicity was found after 72 h. According to MTT assay, the mag.SLPs had the same cytotoxicity as the SLPs, IO-OA, and IO at concentration of 1 μg/ml (Figures 5A,B). When the concentration was increased 10-fold, both mag.SLPs and SLPs showed similar cytotoxicity, which was significantly higher than that for IO-OA and IO. In Jurkat cells treated with the particles for 24 h, *IC*_50_ for the mag.SLPs was 3.8 μg/ml, while *IC*_50_ for the SLPs, IO-OA, and IO was >10 μg/ml (Table 2). *IC*_50_ for the mag.SLPs was 0.9 μg/ml for Jurkat cells treated with the particles for 72 h, while *IC*_50_ for the SLPs was 10 μg/ml, and *IC*_50_ for the IO-OA and IO was >10 μg/ml (Table 2). Dox demonstrated more pronounced toxicity than the mag.SLPs, SLPs, IO-OA, and IO toward Jurkat cells after both 24 and 72 h of treatment (Table 2; Figures 5E,F).

Also HL-60/wt cells were sensitive to cytotoxic effect of the particles. In the trypan blue exclusion test, the particles at concentration of 1 μg/ml showed similar cytotoxicity for Jurkat and HL-60/wt cells at both time points, i.e., 24 and 72 h (Figure 4). However, the mag.SLPs and SLPs at concentration of 10 μg/ml showed more pronounced anticancer activity than the IO-OA, and IO toward HL-60/wt cells (Figures 4C,D). Similarly to results with Jurkat cells, cytotoxicity of the mag.SLPs and SLPs at the highest concentration toward HL-60/wt cells measured by the MTT assay was significantly higher compared to that of the IO-OA and IO nanoparticles; a minor anticancer effect was observed at concentration of 1 μg/ml (Figures 5C,D). *IC*_50_ for both mag.SLPs and their components was higher than the *IC*_50_ for Dox toward HL-60/wt cells, however, this difference was not as significant as that for Jurkat cells. After 24 h of incubation with HL-60/wt cells, *IC*_50_ for the mag.SLPs, SLPs, IO-OA, and IO was 2.7, 3.5, 3.5, and 0.9 μg/ml, respectively (Table 2). *IC*_50_ for the mag.SLPs treated with HL-60/wt cells for 72 h was 2.3 μg/ml, *IC*_50_ for the SLPs, IO-OA, and IO was 3.3, 1.2, and 0.9 μg/ml (Table 2).

**Figure 6:**
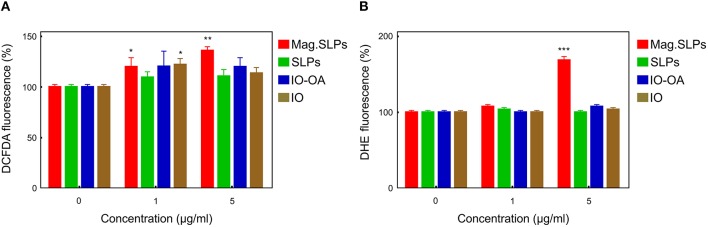
Flow cytometric analysis of ROS level in Jurkat cells treated with mag.SLPs, SLPs, IO-OA, and IO for 24 h. **(A)** Cell staining with 2′,7′-dichlorofluorescein diacetate (DCFDA; H_2_O_2_ detection) and **(B)** dihydroethidium (DHE; O${}_{2}^{-}$ detection). *p < 0.05, **p < 0.01, and ***p < 0.001 compared to non-treated control cells.

Conventional flow cytometry was used for measuring ROS level in the Jurkat cells treated with all four types of the particles. The technique is based on fluorescent dyes, DCFDA and DHE, for detecting H_2_O_2_ and superoxide anion radicals (O_2_^−^), respectively. When Jurkat cells were treated with the mag.SLPs, SLPs, IO-OA, and IO particles, only the mag.SLPs induced both H_2_O_2_ and O_2_^−^ production (Figure 6). A slight increase in DCFDA fluorescence, corresponding to higher concentration of H_2_O_2_, was observed at both concentrations, i.e., 1 and 5 μg of the particles/ml, especially for the mag.SLPs (Figure 6A). In contrast, upregulated DHE fluorescence, attributed to growing O_2_^−^ concentration, was found only for the mag.SLPs at a high concentration (Figure 6B). H_2_O_2_ and O_2_^−^ levels in the mag.SLPs-treated human leukemia cells of Jurkat line were increased by ~20–40 and ~70%, respectively.

**Figure 7:**
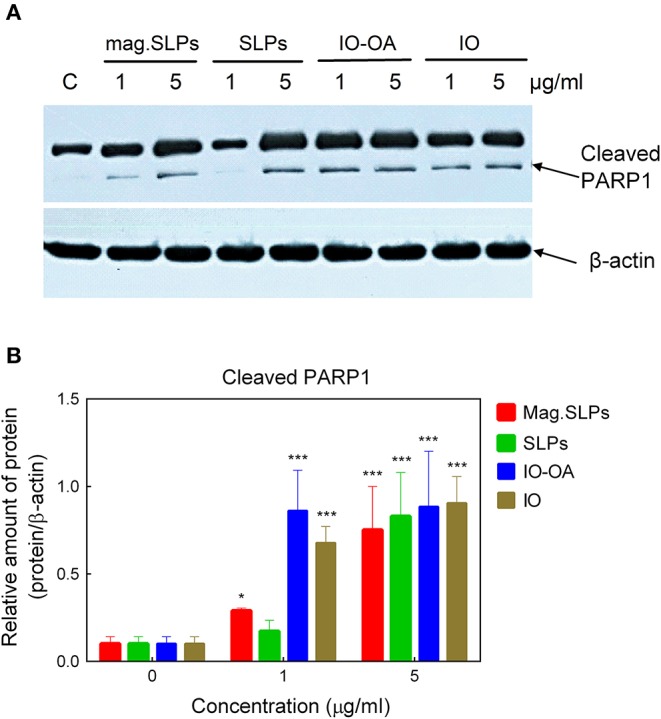
**(A)** Western blot analysis of the level of cleaved apoptosis-associated PARP1 protein in Jurkat cells treated with mag.SLPs, SLPs, IO-OA, and IO particles (1 and 5 μg/ml), C - non-treated control cells. **(B)** Densitometry of protein amount in cells treated with particles (1 and 5 μg/ml). *p ≤ 0.05 and ***p ≤ 0.001 compared to non-treated control cells.

Results of Western blot analysis demonstrated that mag.SLPs, IO-OA, and IO at both concentrations, and SLPs at 5 μg/ml induced an increase in the amount of cleaved form of PARP1 (Figures 7A,B). Appearance of cleaved PARP1 is considered to be an indicator of apoptosis (Brustmann, 2007).

**Figure 8:**
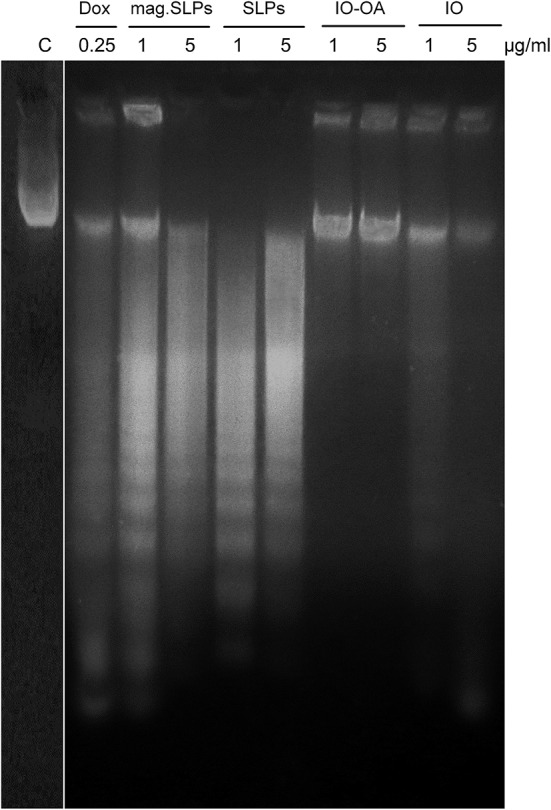
DNA ladder (electrophoresis in 1.5% agarose gel) of Jurkat cells treated for 24 h with doxorubicin (Dox; 0.25 μg/ml), mag.SLPs, SLPs, IO-OA, and IO (all particles at 1 and 5 μg/ml). C - non-treated control cells.

At electrophoresis of DNA from Jurkat cells in the agarose gel, typical DNA laddering caused by the inter-nucleosomal fragmentation was revealed in cells treated with the mag.SLPs and SLPs (Figure 8). Increase of particle doses from 1 to 5 μg/ml led to a decrease in the amount of the apoptotic DNA and increase in the amount of the necrotic DNA. Both apoptotic and necrotic DNA was detected after cell treatment with doxorubicin. There was no significant DNA fragmentation in cells treated with IO and IO-OA (1 and 5 μg/ml). Thus, the mechanism of Jurkat cell death (apoptosis *vs*. necrosis) induced by the mag.SLPs and SLPs depends significantly of the dose of the particles.

To study DNA fragmentation at the induced cell death, DNA comet assay characterizing single-strand breaks of DNA was used under alkaline conditions. The mag.SLPs and SLPs dose-dependently induced the DNA damage events in Jurkat cells, while such effects of the IO and IO-OA were significantly less pronounced (Figures S1, S2). It should be noted that at 5 μg/ml, DNA damaging effect of the mag.SLPs and SLPs was comparable with that of doxorubicin (0.25 μg/ml).

**Figure 9:**
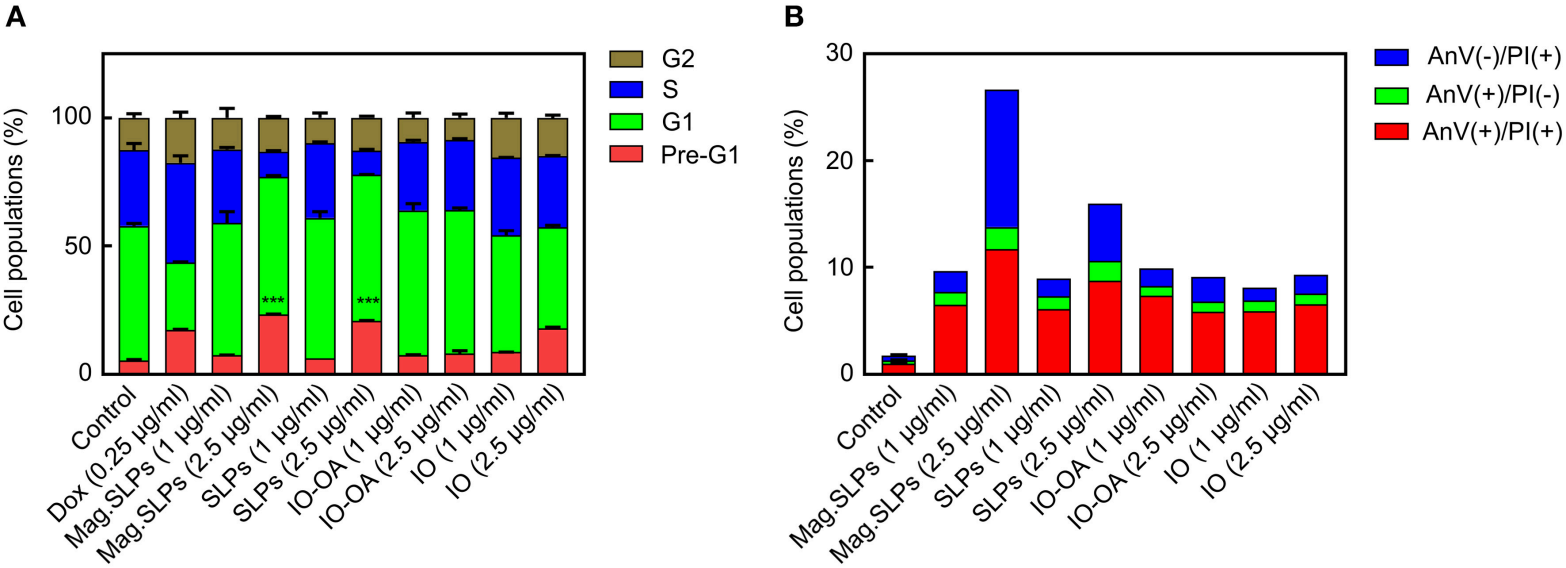
Cell cycle arrest and cell death induction by mag.SLPs. **(A)** Dose-dependent impact of the mag.SLPs, SLPs, IO-OA, and IO on the distribution of Jurkat T-leukemia cells in different phases of the cell cycle determined by FACS analysis of propidium iodide (PI)-stained cells after 24 h of continuous exposure. **(B)** Impact of the particles after 24 h of incubation on the phosphatidylserine externalization in Jurkat T-leukemia cells measured by FACS analysis using FITC-labeled Annexin V and PI staining. Dox, doxorubicin (positive control). ***p ≤ 0.001 compared to control untreated cells.

To reveal the mechanism of cell death induction by the mag.SLPs, FACS analysis was used with PI staining of the leukemia cells treated with 1 and 2.5 μg of mag.SLPs and SLPs per ml and 1 and 5 μg of IO-OA and IO per ml (Figure 9A; Figure S3 and Table S1). Mag.SLPs, SLPs, IO-OA, and IO at concentration of 1 μg/ml were relatively non-toxic for Jurkat T-leukemia cells and they did not exhibit a significant impact on cell cycle progression, in contrast to doxorubicin (positive control; 0.25 μg/ml) that caused a distinct S/G_2_ block in the cells. At high dose (5 μg/ml), the mag.SLPs demonstrated a strong cytotoxic activity with >76% of Jurkat T-leukemia cells in a pre-G1 phase (apoptosis). At the same concentration, the SLPs killed 69% of the cells and IO-OA or IO (5 μg/ml) did not induce cytotoxicity, suggesting that only mag.SLPs were effectively killing tumor cells (Table S1).

Annexin V/PI double staining confirmed cell cycling data (Figure 9B; Figure S4). Microparticles induced early [Annexin V(+)/PI(–)] and late apoptosis [Annexin V(+)/PI(+)]. However, an increase of the mag.SLPs and SLPs concentration to 2.5 μg/ml was accompanied by the elevation of the amount of the necrosis cells [Annexin V(–)/PI(+)]. Results at higher concentration (5 μg of mag.SLPs or SLPs per ml) were not presented due to a massive late apoptosis revealed by both AnV externalization, rupture of cell membrane, and necrosis of the treated leukemia cells.

**Figure 10:**
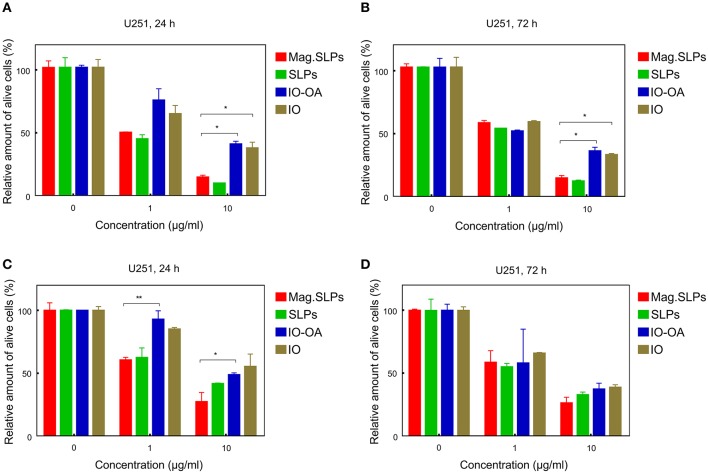
Cytotoxicity of mag.SLPs, SLPs, IO-OA, and IO particles against human glioblastoma U251 cells determined by **(A,B)** trypan blue exclusion test and **(C,D)** MTT assay after **(A,C)** 24 and **(B,D)** 72 h of treatment. *p ≤ 0.05 and **p ≤ 0.01 compared to the mag.SLPs.

In addition to T leukemia Jurkat and human leukemia HL-60/wt cells, also U251 human glioblastoma cells were treated with the particles for 24 and 72 h and cell viability was measured by the trypan blue exclusion and MTT assays (Figure 10). In contrast to Jurkat and HL-60/wt cells, U251 cells grow in a monolayer culture and not in a suspension. In general, anticancer effects of the particles measured by both trypan blue exclusion test and MTT assay showed a similar pattern (Figure 10). At concentration of 1 μg/ml, the IO-OA and IO demonstrated relatively weak cytotoxicity *in vitro* toward glioma cells after 24 h of treatment, while 72 h of incubation with the mag.SLPs, SLPs, IO-OA, and IO resulted in comparable anticancer effects. At 10-fold higher concentrations, mag.SLPs and SLPs possessed higher cytotoxicity toward the glioblastoma cells (*IC*_50_ = 1.2 and 1.1 μg/ml, respectively; Table 2) than the IO-OA and IO (*IC*_50_ = 7.4 and 5.9 μg/ml, respectively). For U251 cells treated with the mag.SLPs, SLPs, IO-OA, and IO for 72 h, *IC*_50_ was 2.3, 1.6, 1.3, and 3.4 μg/ml (Table 2). The indicator of cytotoxicity (*IC*_50_) of Dox for U251 glioma cells (24 h treatment) was comparable (1.7 μg/ml) with that for the mag.SLPs (1.2 μg/ml) and the SLPs (1.1 μg/ml).

**Figure 11:**
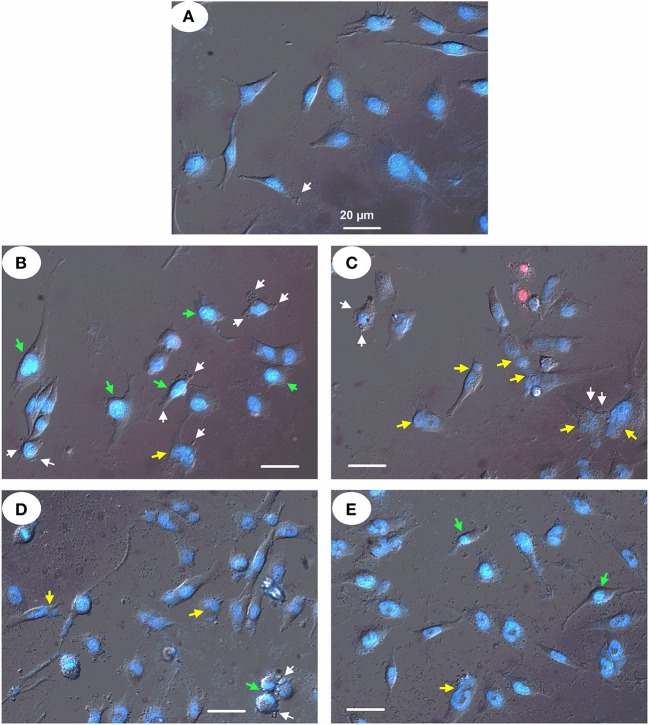
Micrographs of human glioblastoma U251 cells treated with **(B)** mag.SLPs, **(C)** SLPs, **(D)** IO-OA, and **(E)** IO particles for 24 h; **(A)** control. Differential interference contrast image of treated cells stained with fluorescent DNA-specific Hoechst-33342 (blue) and fluorescent DNA/RNA-specific PI (red). Yellow, green, and white arrows indicate chromatin fragmentation, its condensation, and plasma membrane blebbing, respectively. Bar corresponds to 20 μm.

Finally, fluorescent microscopy of glioma U251 cells treated with the particles after double staining of chromatin with Hoechst 33342 (blue fluorescence) and PI (red fluorescence) revealed cells with changed shape and morphology of the nucleus. This was documented by increased condensed chromatin, nuclear fragmentation, and membrane blebbing (Figure 11). The mag.SLPs caused higher chromatin condensation of U251 cells, while the SLPs induced more chromatin fragmentation. The IO-OA and IO induced only some chromatin fragmentation and condensation of U251 cells indicating predominantly apoptosis induction.

## Discussion

The magnetic component of the temperature-responsive solid-lipid particles was based on coprecipitated IO nanoparticles that are polydispersed, spherical-like in shape and with size depending on parameters such as temperature, stirring rate, type of iron salts, etc. (Wu et al., 2008). Particles with diameters close to those synthesized in this study (*D*_n_ = 10 nm) have been already reported by other authors (Salazar et al., 2011; Fang et al., 2012; Mascolo et al., 2013). Significant difference between *D*_n_ and *D*_h_ (150 nm) resulted from several factors, including contribution of solvation layer to *D*_h_ value, lack of steric stabilization leading to agglomeration, and sensitivity of DLS to presence of large particles. Relatively low ζ-potential (14 mV) indicated poor stability of IO in water. The minor weight loss in TGA was attributed to evaporation of residual water. In general, magnetic properties of nanoparticles are strongly related to their chemical composition, size, morphology, and crystallinity (Li Q. et al., 2017). With decreasing size, multi-domain structures become single-domain and then, after reaching the critical diameter, superparamagnetic. Superparamagnetism is a desirable property of the nanoparticles intended for a biomedical application, as they do not aggregate in the absence of external magnetic field. However, the particle separation by a magnet is not affected. Negligible remanent magnetization and coercivity suggested that IO is a mixture of superparamagnetic and ferrimagnetic particles (>12.5 nm) (Kafrouni and Savadogo, 2016). Saturation magnetization of the IO nanoparticles was lower than that of bulk IO (*M*_s_ = 76 and 92 A · m^2^/kg for γ-Fe_2_O_3_ and Fe_3_O_4_, respectively) (Berkowitz et al., 1968), but sufficient enough to provide excellent manipulation by a magnetic field. Deterioration of magnetic parameters, especially saturation magnetization, could be caused by mixed chemical composition of various oxides, but also by the presence of antiphase domain boundary structural defects (Nedelkoski et al., 2017).

Successful coating of IO with OA was confirmed by several techniques; e.g., presence of a halo layer around the nanoparticles by a TEM micrograph, increase in *D*_h_ by DLS, and presence of carbon in the IO-OA particles by elemental analysis. Increase of *PDI* indicated aggregation of the particles and limited ability of OA to stabilize them in water. However, it should be noted that the primary intention of introduction of OA layer was to stabilize IO particles in DCM and not in water. Moreover, OA layer could be partially damaged during transfer of the IO-OA particles from organic to aqueous phase via ethanol/water mixtures. Amount of OA in the IO-OA particles calculated from TGA and elemental analysis was 7.1 and 3.3 wt.%, respectively. Moreover, lower saturation magnetization of IO-OA compared to IO particles (by 3.3 A · m^2^/kg) was also caused by the presence of OA shell on the particle core. By comparing saturation magnetizations of hydrophilic IO and hydrophobic IO-OA, amount of OA coating was calculated to 6.1 wt.%.

In the mag.SLPs, TD formed the lipid matrix, carrying the IO-OA to render the particles with magnetic properties. Good compatibility between lipid matrix and IO-OA ensured homogenous distribution of the magnetic nanoparticles within the SLP bulk, which is an important factor to prevent burst release and possible local toxicity that is unfavorable either therapeutically or economically (Huang and Brazel, 2001). Optionally, acceleration of matrix melting, and thus release of the IO-OA in the target location, can be achieved by local heating produced by exposition to AMF. Biocompatible and biodegradable BL (Trousil et al., 2019; Urbánek et al., 2019) was used to stabilize the SLPs and simultaneously to provide a coating that is capable to reduce prospective engulfment by the reticuloendothelial system. Surface modification of particles with poly(ethylene oxide) is a well-established approach to effectively prolong their systemic circulation by limiting particle aggregation, opsonization, and uptake via phagocytosis (Suk et al., 2016).

Both the SLPs and mag.SLPs were characterized by similar *D*_h_, indicating that incorporation of IO-OA nanoparticles into lipid matrix did not affect the size of the lipid particles, however, ζ-potential of the mag.SLPs was slightly lower. Similarly to results obtained for IO nanoparticles, a significant difference was noted between *D*_h_ and *D*_n_ determined from SEM micrographs (854 *vs*. 70–90 nm), which can be caused by the same reasons as described above. Both the size and ζ-potential of the particles are important parameters determining their delivery and biodistribution. The enhanced permeability and retention (EPR) effect, originating from leaking vascularization of tumors, is considered as one of the important factors that can ensure high accumulation of intravenously administered therapeutic agents in the tumor, compared to non-cancerous tissues. The particle size limit for EPR is determined by cut-off pore size of tumor blood vessels, which is usually in the range 380–780 nm (Bae and Park, 2011). As the size of the mag.SLPs approaches the upper limit, it can be assumed that the passive transport of the particles in the tumor tissue will be difficult, but not impossible. Decrease in *D*_h_ of the mag.SLPs could be achieved by additional surface modifications that would improve their stabilization (e.g., by steric repulsion) or additional optimization of the synthesis. In addition, enhanced accumulation of the mag.SLPs in tumors can be achieved by exploiting external AMF and magnetic targeting (Mody et al., 2014).

By comparing saturation magnetization of the starting IO-OA (49.8 A · m^2^/kg) and that of the mag.SLPs (3.6 A · m^2^/kg), the content of IO-OA in the mag.SLPs was calculated to 7.2 wt.%, which is in agreement with both IO amount loaded in the reaction mixture (7.4 wt.%) and Fe content according to AAS (5.6 wt.% Fe represents 7.7 wt.% Fe_3_O_4_). It was only slightly higher than IO amount (5.2 wt.%) estimated from reduction of carbon content in the mag.SLPs compared to the non-magnetic SLPs. It should be noted that the presence of carbon (80 wt.%) in the SLPs was attributed to the lipid matrix and BL (20 wt.%). In the mag.SLP, the heating efficiency, and thus ability to boost the degradation of lipid matrix, was directly related to the contribution of magnetic phase as it is the source of heat. In general, heating under the applied AMF is a result of transformation of magnetic energy through the Brownian motion and Néel relaxation into thermal energy (Kaur et al., 2016). The linear part of the plot measured at 30°C represented stable temperature conditions of the mag.SLP dispersion before the field application (Figure 2e). After 30 s, the magnetic field was switched on and the temperature immediately increased. The heating rate *dT/dt* was determined from the time evolution *vs*. temperature at six different *H* as a linear fit between 40 and 60 s (Figure 2d, black line at *H* = 7.9 kA/m). As *H* increased, Δ*T/*Δ*t* increased too (Figure 2e). The curve represented experimental data fitted by function Δ*T/*Δ*t* = *(H/a)*^*n*^, where *a* and *n* are fitting parameters (Périgo et al., 2013; Skumiel et al., 2013). Observed type of *H*^*n*^ heating rate, where 2 ≤ *n* ≤ 3, is typical for magnetic nanoparticles intended for magnetic hyperthermia applications.

Most of the existing anticancer drugs suffer from adverse effects based on the unspecific effects against normal tissues and organs due to low selectivity and rapid (6–12 months). Moreover, loss of effectiveness induced by development of drug resistance in tumor cells is a major limitation for successful therapy or even cure by chemotherapeutic interventions. One of the possible approaches to increase the selectivity of nanocarriers in cancer therapy is their surface modification with tumor specific targeting moieties that interact with receptors or ligands overexpressed in cancerous cells, e.g., transferrin receptor, folate receptors, or integrin ligands (Bahrami et al., 2017). However, in practice this strategy often fails, mainly due high heterogeneity of tumor cell being at different levels and stages of differentiation, stemness or proliferation (Suk et al., 2016). Consequently, anticancer remedies are needed with low general toxicity in the organism and capability to circumvent mechanisms of cellular resistance to the anticancer agents. Cytotoxicity of chemotherapeutics, including anthracyclines, platinum complexes, and topoisomerase inhibitors, is known to be related to the overproduction of ROS that causes death of cells (Yokoyama et al., 2017; Gorini et al., 2018; Yang et al., 2018). In contrast to anticancer drugs commonly used within systemic therapy, the mag.SLPs are deprived of any medicinal payload and the only source for ROS production are IOs.

For all types of investigated cells, the cytotoxicity was dose-dependent, however, not equal for specific components. It indicates differences in behavior and cellular response to IO particles and SLP system, which can originate from a gradual release of IO from the lipid matrix. High cytotoxicity for human myeloid leukemia HL-60/adr and HL-60/vinc cells resistant to Dox and vincristine, respectively, indicates low vulnerability toward multidrug resistance mechanisms and a clear advantage of the mag.SLPs. The cytotoxicity of the mag.SLPs was higher than that of Dox, which suggests that the mag.SLPs might represent an effective alternative for treatment of tumor cells resistant to traditional anticancer medicines. It is important to note that hyperthermic effect of the mag.SLPs induced by an alternating magnetic field has to be distinguished from the cytotoxicity of particles. As the hyperthermic effect itself kills the cells, there is no sense to determine particle cytotoxicity by MTT assay and/or trypan blue exclusion test in the presence of magnetic field that would interfere with the particle cytotoxicity determination.

To address the mechanism responsible for the anticancer effects of the developed particles, two parameters were studied: (*i*) ROS (O${}_{2}^{\cdot -}$ radicals and H_2_*O*_2_) production and (*ii*) pro-apoptotic changes in cell morphology induced by the mag.SLPs, SLPs, IO-OA, and IO particles in human tumor cells, i.e., leukemia T-cells of Jurkat line. Due to the fact that lipid matrix is the only protection of IO nanoparticles after release, their surface is exposed to the action of many components, including H_2_O_2_ that is produced by superoxide dismutase from O${}_{2}^{\cdot -}$ via disproportion reaction (Sun et al., 2018). It was reported that Fe^2^+^^ or Fe^3^+^^ ions react with H_2_*O*_2_ via Fenton reaction, generating hydroxyl radicals (OH^·^) that can be extremely effective in anticancer therapy (Eguchi et al., 2015); their concentration correlates with O${}_{2}^{\cdot -}$ amount. It should be mentioned that O${}_{2}^{\cdot -}$ radicals are more damaging to normal tissues and organs than H_2_*O*_2_ (Phaniendra et al., 2015). A strong cytotoxic activity of the mag.SLPs (Figures 4, 5) seems directly correlate with their higher capability to induce ROS generation (Figure 6). A significantly increased O${}_{2}^{\cdot -}$ level was observed only for the mag.SLPs, while enhanced H_2_O_2_ concentration was found for each type of the tested particles, though not all results were statistically significant. This could be associated with lower particle concentration (5 μg/ml) used in ROS analysis than maximal concentration (10 μg/ml) in the viability assays. Earlier, we have found that Dox (0.5–1 μM) did not affect significantly H_2_O_2_ level in Jurkat cells and the ROS level increased ~2-fold only after 24 h of treatment (Panchuk et al., 2017). At the same time, O${}_{2}^{\cdot -}$ level increased after 24 h of treatment with Dox 2.5-fold compared to the control untreated cells (Panchuk et al., 2017). It should be noted that, while formation of H_2_O_2_ by Dox was rather late, after 24 h of treatment, O${}_{2}^{\cdot -}$ radical induction was detected already after 3 h of Dox action. These results are in agreement with literature data, where the magnetic iron oxide nanoparticles increased ROS level in various cell lines *in vitro* and in animal models (Mahmoudi et al., 2011), e.g., polyethyleneimine or poly(acrylic acid)-coated magnetic iron oxide nanoparticles decreased the maternal weight gain and increased fetal deaths in pregnant CD-1 mice (Di Bona et al., 2014). The mag. SLPs and non-mag. SPLs exhibited comparable cytotoxicity toward cancer cells (Figures 4, 5). However, the mag. SLPs at a high concentration (5 μg/ml) induced a significant increase in the O${}_{2}^{\cdot -}$ level in Jurkat cells (Figure 6), which was not found with non-mag. SPLs.

Cleavage of PARP1 (reparation enzyme) was detected by Western blot analysis of proteins of Jurkat cells treated with the particles, some of which have demonstrated the cytotoxic action. An increased level of the cleaved form of PARP1 was detected for mag.SLPs, SLPs, IO-OA, and IO particles, although most of PARP1 protein remained uncleaved. Typically, DNA breaks induce poly(ADP-ribosyl)ation via activation of poly(ADP-ribose) polymerase (PARP) (Zhang and Xu, 2000; Brustmann, 2007; Li Z. H. et al., 2017), which causes a depletion of NAD+ and ATP, resulting in the induction of necrosis (Herceg and Wang, 1999). However, point mutation in the cleavage site of PARP, which leads to its resistance to caspases, accelerated cell death induced by the tumor necrosis factor alpha. Thus, PARP cleavage prevented necrosis during induction of apoptosis. PARP-deficient lymphocytes, fibroblasts, hepatocytes, and neuronal cells demonstrated normal apoptosis induced by different agents, which suggests that PARP might be not important in the apoptotic events (Leist et al., 1997; Wang et al., 1997). DNA fragmentation did not increase cell death, suggesting that it might be a downstream event (Herceg and Wang, 1999). It was speculated that PARP cleavage is a mechanism ensuring the normal speed and order of apoptotic events and protection of cells from necrotic death (Herceg and Wang, 1999). As a result, cytotoxicity of the mag.SLPs and SLPs microparticles toward Jurkat T cells might be associated with the DNA fragmentation, however, in case of IO-OA and IO particles, PARP1 cleavage was observed in Jurkat cells without a significant DNA fragmentation. Possible reasons for such discrepancy were discussed above.

FACS analysis of the pattern of cell cycle arrest demonstrated a significant increase in number of Jurkat cells in the pro-apoptotic (pre-G_1_) phase under the effect of the mag.SLPs and SLPs. This cytotoxic effect was dose-dependent and expressed only at 5 μg of particles per ml; it was not significant at 1 μg/ml. However, FACS analysis of the pattern of cell death induction based on measuring Annexin V externalization and PI staining of cells with ruptured membrane suggested that cell necrosis was also involved in the action of the mag.SLPs and SLPs. As predicted, IO-OA and IO did not induce cell death at both concentrations (1 and 5 μg/ml).

Our studies demonstrated an induction of death of Jurkat leukemia cells by the mag.SLPs and SLPs microparticles, while the IO-OA and IO particles were much less cytotoxic. While at low dose of the mag.SLPs and SLPs (1 μg/ml), apoptosis was better expressed (DNA laddering and DNA comet analyses), signs of cell necrosis were also revealed at high dose (5 μg/ml). Western blot analysis revealed that the reparation enzyme PARP1 was partially cleaved under the action of all types of the particles used. In order to clear up the situation, one more indicator of cell death was used, i.e., FACS measurement of AnV(+)/PI(–) ratio (pro-apoptotic cells), AnV(+)/PI(+) ratio (pro-necrotic cells), and the amount of pro-apoptotic (pre-G1) phase cells. Decreasing dose of the mag.SLPs and SLPs also reduced the amount of the necrotic cells. It is known that both apoptosis and necrosis are the main mechanisms of cell death induced by different agents. However, apoptosis or necrosis induction strongly depends on dose and/or duration of action of the cytotoxic agent. Thus, there are no “pure” apoptosis or necrosis inducers, and usually, more prolonged action of apoptosis inducer or its action in higher dose can cause secondary necrosis of the treated cells. Some differences in the revealed ratio of the apoptotic to necrotic Jurkat cells treated with the particles might be caused by evaluating different apoptotic events, namely phosphatidylserine externalization on cell surface (Annexin V staining) or the fragmentation of DNA in cell nucleus (DNA laddering due to inter-nucleosomal cleavage and appearance of DNA comets due to single strand breaks).

Another mechanism proposed for the cytotoxic action of the IO particles in biomedical applications might be their interfering with the actin cytoskeleton that resulted in inhibition of cell proliferation (Soenen et al., 2009). A disruption of cytoskeleton proteins, such as tubulin and dynamic cortical meshwork of F-actin, was induced under the action of the magnetic nanoparticles (Berry et al., 2004; Radu et al., 2010; Boraschi et al., 2012). Besides, changes in cellular behavior were accompanied with an elevated expression of specific cytokines (IL-1, IL-4, and IL-10), while inhibition of TNF-α suggested a potential effect of the magnetic nanoparticles on the immune responses (Naveau et al., 2006; Siglienti et al., 2006; Hsiao et al., 2008).

When studying the morphological impairments in human U251 glioma cells treated with the particles, the pro-apoptotic changes in cell shape and nucleus morphology, i.e., condensed chromatin, nuclear fragmentation, and plasma membrane blebbing, were detected after double staining of nuclear chromatin with Hoechst 33342 and PI. We have found that the mag.SLPs and SLPs were highly toxic for human leukemia T cells of Jurkat line. Both apoptosis- and necrosis-related mechanisms might be responsible for the cytotoxic action of the mag.SLPs and SLPs toward Jurkat (suspension) and glioblastoma (monolayer) cells.

## Conclusions

In summary, the developed mag.SLPs demonstrated promising characteristics suggesting application for anticancer therapy especially in the context of therapy resistance. While tumor cells growing in the monolayer can be treated locally, leukemia cells growing in a suspension can be killed due to relatively large size of the mag.SLPs (~0.8 μm). Prospectively, an additional biofunctionalization of the mag.SLPs with drugs can be considered for application in recirculating perfusion systems. Moreover, magnetically based selection of targeted tumor cells will be possible to achieve an addressed response of the SLPs for extracorporeal clearance of cells or toxic compounds from blood of patients suffering from the oncohematological diseases (e.g., leukemia) or autoimmune disorders (e.g., systemic *lupus erythematosus*). Cytotoxicity might be induced by both the lipid shell and the IO core, which separately proved to be relatively non-toxic, demonstrating cytotoxicity only in high doses (5–10 μg/ml). A synergic contribution of these components in the here described cytotoxicity is possible. Thus, the system provides highly effective, functional and selective delivery of superparamagnetic IO nanoparticles in the cells, maximally generating cytotoxic ROS by mechanism widely not vulnerable by multidrug resistance. Cytotoxicity of the mag.SLPs, SLPs, IO-OA, and IO particles toward treated Jurkat T cells might be associated with the induction of the pro-apoptotic DNA fragmentation. The results demonstrated that the mag.SLPs and SLPs were highly toxic for human leukemia T cells of Jurkat line and the mechanisms of cell death induction included both the apoptosis (expression of apoptosis-related proteins, internucleosomal DNA fragmentation, and cell cycle distribution) and the necrosis (Annexin V-stained phosphatidyl serine externalization in plasma membrane and membrane rupture detected via PI staining) of the treated cells. It takes advantage of thermoresponsiveness of the SLPs, which can melt down in the tumorous tissue that is inherently hyperthermic and at the same time is accessible to high frequency magnetic field. Thus, the system represents a perspective theranostic combining a chemotherapy and diagnostics (e.g., MRI and magnetic particle imaging), which will be more effective than IO or carrier (SLPs).

## Data Availability Statement

The datasets generated for this study are available on request to the authors Daniel Horák (horak@imc.cas.cz) and Rostyslav Stoika (stoika.rostyslav@gmail.com).

## Author Contributions

MŚ synthesized and characterized the particles. RP determined reactive oxygen species and FACS analysis. NS prepared cells cultures and studied cytotoxicity. PC prepared the solid-lipid particles. NF performed MTT tests, Western blot analysis and DNA ladder and comet assays. OK performed trypan blue exclusion test, microscopy study and DNA comet assay. MH designed solid-lipid experiment. MM measured heat dissipation. WB provided cell cultures. JT synthesized poly(ethylene oxide)-*block*-poly(ε-caprolactone). RS designed biological experiments. DH wrote the manuscript.

### Conflict of Interest

The authors declare that the research was conducted in the absence of any commercial or financial relationships that could be construed as a potential conflict of interest.
