# Supplementary material for: Magnetic Temperature-Sensitive Solid-Lipid Particles for Targeting and Killing Tumor Cells
Source: Front Chem. 2020 Apr 9;8:205. doi: 10.3389/fchem.2020.00205 (PMC7161697; doi:10.3389/fchem.2020.00205)
Supplement: Supplementary file 1 [file Data_Sheet_1.docx]

**Supplementary Materials**

**Table S1.** Changes in cell cycle distribution in Jurkat T-leukemia cells incubated with mag.SLPs, SLPs, IO-OA, and IO particles.

| Sample | Number of cells in each cell cycle phase (mean ± SD) | | | |
| --- | --- | --- | --- | --- |
|  | Pre-G_1_ | G_1_ | S | G_2_/M |
| Control | 5.58±0.26 | 52.44±0.84 | 29.4±2.67 | 12.58±1.57 |
| Dox (0.25 µg/ml) | 17.41±0.22** | 26.18±0.29 | 38.95±2.74** | 17.47±2.23 |
| Mag.SLPs (1 µg/ml) | 7.61±0.04 | 51.47±4.39 | 28.64±0.76 | 12.27±3.67 |
| Mag.SLPs (2.5 µg/ml) | 23.47±0.15*** | 53.57±0.51 | 9.92±0.25 | 13.04±0.61 |
| Mag.SLPs (5 µg/ml) | 76.69±2.30** | 13.81±1.72 | 6.63±0.81 | 2.87±0.22 |
| SLPs (1 µg/ml) | 6.39±0.22 | 54.65±2.44 | 29.14±0.53 | 9.82±1.91 |
| SLPs (2.5 µg/ml) | 21.01±0.12*** | 56.89±0.03 | 9.33±0.50 | 12.78±0.65 |
| SLPs (5 µg/ml) | 69.35±0.38** | 19.01±0.64 | 7.87±0.15 | 3.76±0.41 |
| IO-OA (1 µg/ml) | 7.69±0.08 | 56.30±2.63 | 26.60±0.77 | 9.41±1.95 |
| IO-OA (5 µg/ml) | 8.23±1.13 | 55.83±0.83 | 27.39±0.45 | 8.55±1.51 |
| IO (1 µg/ml) | 8.75±0.06 | 45.52±1.81 | 30.28±0.05 | 15.44±1.81 |
| IO (5 µg/ml) | 17.99±0.55* | 39.51±0.68 | 27.78±0.09 | 14.73±1.13 |

Dox – doxorubicin, control - cells in the absence of particles, * р <0.01 relative to control, ** р <0.001 relative to control, *** р <0.001 relative to control.





**Figure S1.** Micrographs of DNA comet assay in Jurkat cells treated for 24 h with (a) mag.SLPs (1 µg/ml), (b) mag.SLPs (2.5 µg/ml), (c) mag.SLPs (5 µg/ml), (d) SLPs (1 µg/ml), (e) SLPs (2.5 µg/ml), (f) SLPs (5 µg/ml). (g) IO-OA (1 µg/ml), (h) IO-OA (5 µg/ml), (j) IO (1 µg/ml), (k) IO (5 µg/ml), (l) Dox (0.25 µg/ml), and (i) non-treated control cells. The bars on the microphotographs represent 20 μm.





**Figure S2.** Quantitative DNA comet assay in Jurkat cells treated with mag.SLPs, SLPs, IO-OA, and IO. C – non-treated control cells, Dox – doxorubicin. * p ≤0.05, ** p ≤0.01, and *** p <0.001 compared to non-treated control cells.


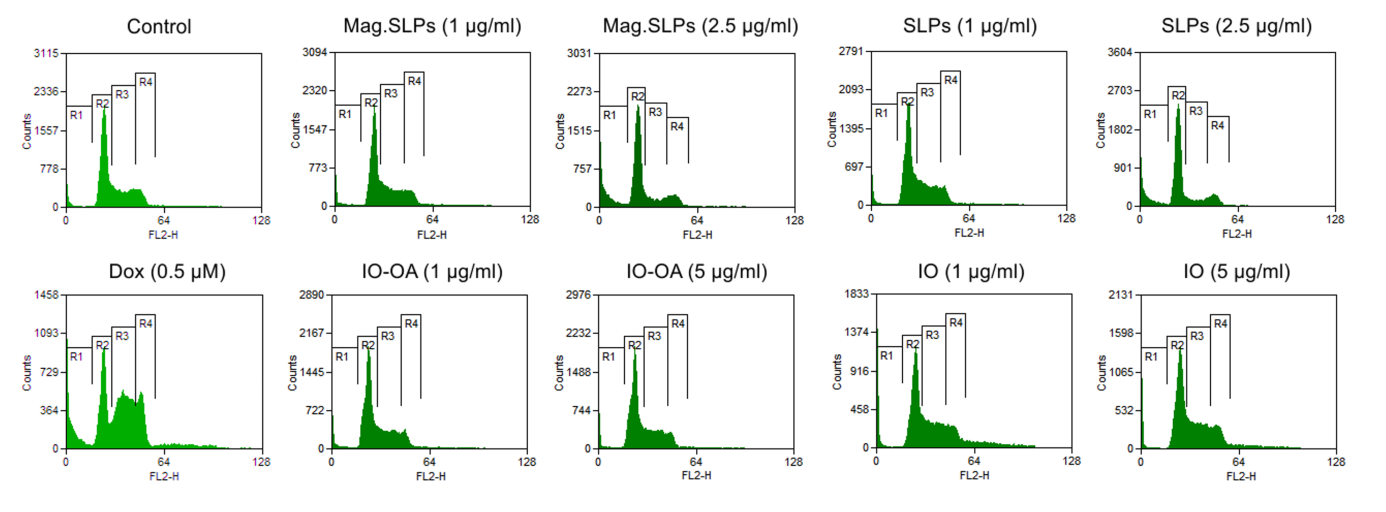


**Figure S3.** Histograms of cell cycle distribution (FACS analysis) in Jurkat T-leukemia cells incubated with mag.SLPs, SLPs, IO-OA, IO, and doxorubicin (Dox). Control - cells in the absence of particles.


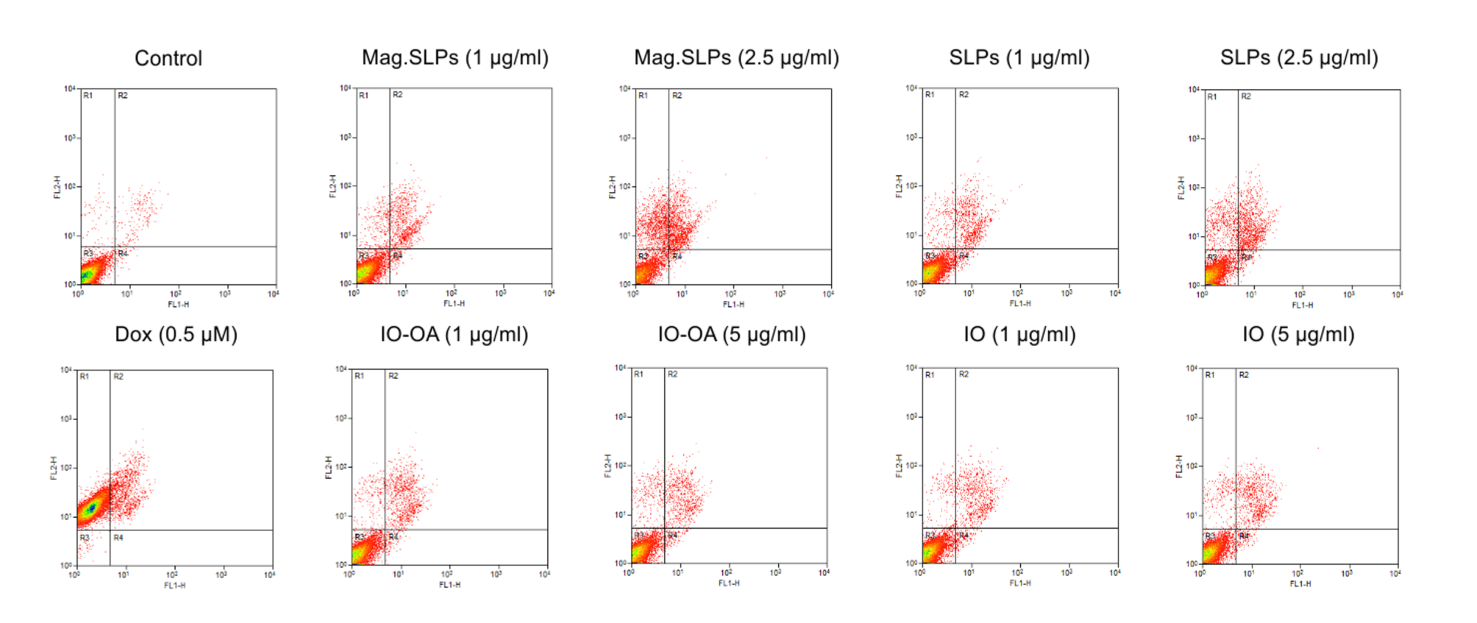


**Figure S4.** Impact of mag.SLPs, SLPs, IO-OA, IO particles, and doxorubicin (Dox; positive control) after 24 h of incubation on phosphatidylserine externalization in Jurkat T-leukemia cells (FACS analysis using FITC-labeled Annexin V and PI staining). Control - cells in the absence of particles.
